# Ticarcillin degradation product thiophene acetic acid is a novel auxin analog that promotes organogenesis in tomato

**DOI:** 10.3389/fpls.2023.1182074

**Published:** 2023-09-04

**Authors:** Suja George, Mohammed Rafi, Maitha Aldarmaki, Mohamed ElSiddig, Mariam Al Nuaimi, Naganeeswaran Sudalaimuthuasari, Vishnu Sukumari Nath, Ajay Kumar Mishra, Khaled Michel Hazzouri, Iltaf Shah, Khaled M. A. Amiri

**Affiliations:** ^1^ Khalifa Center for Genetic Engineering and Biotechnology, United Arab Emirates University, Al-Ain, United Arab Emirates; ^2^ Department of Chemistry, College of Science, United Arab Emirates University, Al Ain, United Arab Emirates; ^3^ Department of Biology, College of Science, United Arab Emirates University, Al Ain, United Arab Emirates

**Keywords:** tomato, timentin, ticarcillin, organogenesis, transcriptome, thiophene acetic acid, auxin, lncRNAs

## Abstract

Efficient regeneration of transgenic plants from explants after transformation is one of the crucial steps in developing genetically modified plants with desirable traits. Identification of novel plant growth regulators and developmental regulators will assist to enhance organogenesis in culture. In this study, we observed enhanced shoot regeneration from tomato cotyledon explants in culture media containing timentin, an antibiotic frequently used to prevent Agrobacterium overgrowth after transformation. Comparative transcriptome analysis of explants grown in the presence and absence of timentin revealed several genes previously reported to play important roles in plant growth and development, including Auxin Response Factors (ARFs), GRF Interacting Factors (GIFs), Flowering Locus T (SP5G), Small auxin up-regulated RNAs (SAUR) etc. Some of the differentially expressed genes were validated by quantitative real-time PCR. We showed that ticarcillin, the main component of timentin, degrades into thiophene acetic acid (TAA) over time. TAA was detected in plant tissue grown in media containing timentin. Our results showed that TAA is indeed a plant growth regulator that promotes root organogenesis from tomato cotyledons in a manner similar to the well-known auxins, indole-3-acetic acid (IAA) and indole-3-butyric acid (IBA). In combination with the cytokinin 6-benzylaminopurine (BAP), TAA was shown to promote shoot organogenesis from tomato cotyledon in a concentration-dependent manner. To the best of our knowledge, the present study reports for the first time demonstrating the function of TAA as a growth regulator in a plant species. Our work will pave the way for future studies involving different combinations of TAA with other plant hormones which may play an important role in *in vitro* organogenesis of recalcitrant species. Moreover, the differentially expressed genes and long noncoding RNAs identified in our transcriptome studies may serve as contender genes for studying molecular mechanisms of shoot organogenesis.

## Introduction

Efficient regeneration of transgenic plants from explants after transformation is often the most crucial step in developing genetically modified plants with desirable traits. A good regeneration protocol that works for one genotype would often require considerable modifications, especially in the case of hormone combinations, for different genotypes of the same species (Altpeter et al., 2016). Plant hormones auxins and cytokinins play important roles in developmental switch, organogenesis, and growth. Standardization of plant regeneration protocols involve determining the best hormone combination for different stages of explant growth and organogenesis. Plants have naturally occurring auxins, including the common indole-3-acetic acid (IAA), as well as other endogenous auxins, such as indole-3-butyric acid (IBA), 4-chloroindole-3-acetic acid (4-Cl-IAA), and phenylacetic acid (PAA). A few synthetic auxin-like substances such as naphthalene-1-acetic acid (NAA), 2,4-dichlorophenoxyacetic acid (2,4-D), and picloram are also widely used as plant growth regulators (PGRs) in plant tissue culture (Ma et al., 2018). Therefore, the identification of new plant growth regulators may contribute in developing new hormonal combinations for the regeneration of recalcitrant species *in vitro*.

At the molecular level, pluripotency of plant somatic cells is controlled by a complex regulatory network of genes (Lian et al., 2022). Recent evidence has shown that genes, known as developmental regulators (DRs), can determine and switch plant cell fate, thereby influencing organogenesis and growth. A few DRs such as WUSCHEL (WUS), AUXIN RESPONSE FACTOR (ARF), GROWTH‐REGULATING FACTORs (GRF), LEAFY COTYLEDONs (LEC1 and LEC2), BABY‐BOOM (BBM), CUP‐SHAPED COTYLEDON (CUC1 and CUC2), CLAVAT3 (CLV3), and ENHANCED SHOOT REGENERATION (ESR), etc., have been extensively studied and are used to enhance organogenesis in various plant species (Debernardi et al., 2020; Lian et al., 2022). However, the use of many of these DRs is restricted by patents, and the identification of new DRs is necessary to encompass a broader range of recalcitrant species and genotypes. Understanding the molecular mechanisms of auxin perception, transport, and signal transduction is essential for identifying key DR genes.

Antibiotics such as timentin, carbenicillin, and cefotaxime which suppress Agrobacterium growth of after co-cultivation are commonly used in plant transformation experiments. Timentin is a combination of ticarcillin, a broad-spectrum semi-synthetic β-lactam antibiotic, and clavulanic acid, a β-Lactamase competitive inhibitor; that confers stability to β-lactam ring-containing antibiotics. Previous studies have reported a positive effect of β-lactam ring-containing antibiotics such as timentin, carbenicillin, and penicillin G on shoot regeneration in species such as tomato, tobacco, and London plane tree. This effect was found to be dependent on the antibiotic concentration and plant genotype (Nauerby et al., 1997; Ling et al., 1998; Costa et al., 2000; Li et al., 2007; Varlamova et al., 2021). In contrast, some studies have also reported no significant effect of these antibiotics on shoot regeneration in species such as tobacco and Siberian elm (Cheng et al., 1998).

**Figure 1:**
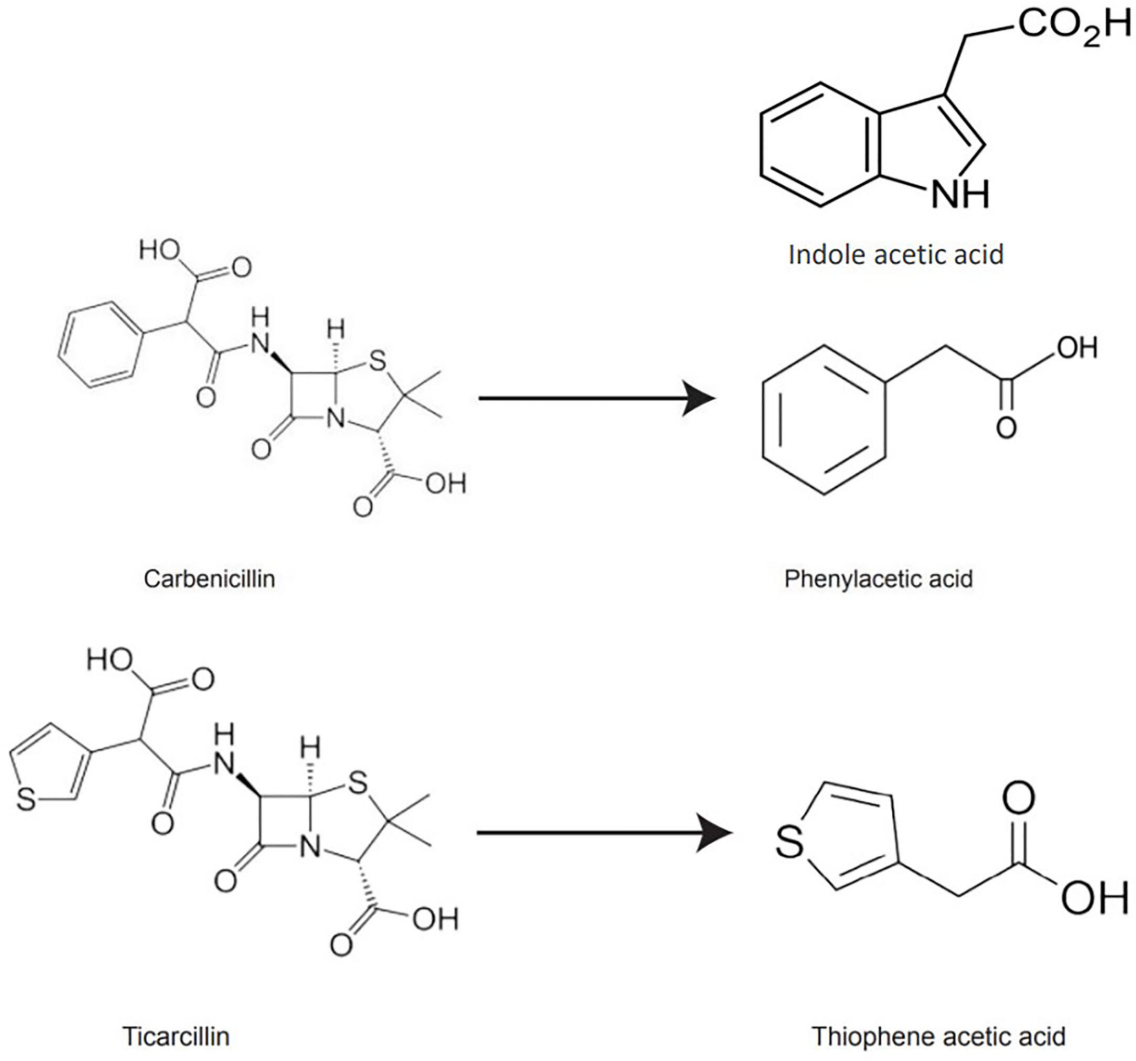
Chemical structures of IAA, carbenicillin, ticarcillin, phenylacetic acid, and thiophene acetic acid.

β-lactam antibiotics have auxin-like structural features, and their degradation products may exhibit activities similar to auxins in the culture medium. PAA, a naturally occurring auxin, was detected as a degradation product of carbenicillin (Holford and Newbury, 1992). Based on ticarcillin’s structure, we hypothesized that thiophene-3-acetic acid (TAA hereafter) could be a potential breakdown product of ticarcillin. TAA is an organosulfur compound (molecular formula C6H6O2S) and exist as two isomers (thiophene-2-acetic acid, thiophene-3-acetic acid). It is possible that the enhanced shoot regeneration in the presence of timentin from other studies is due to the breakdown product TAA, that acts as a plant growth regulator (**Figure 1**).

In this study, we tested the regeneration from tomato (*Solanum lycopersicum L.*) cotyledon explants in culture media containing timentin. To understand the molecular mechanisms by which timentin enhances shoot regeneration in tomato, we performed transcriptome analysis of tomato shoot tissue regenerated in the presence of timentin. Even though previous studies have reported enhanced shoot regeneration in the presence of timentin, to our knowledge; there are no reports available on gene expression patterns specific to the presence of timentin in any species. In addition to protein-coding RNAs, small RNAs, and long non-coding RNAs (lncRNAs), regulate important molecular and biological processes in eukaryotic systems (Wang and Chekanova, 2017). lncRNAs regulate the expression of nearby protein-coding genes (cis-regulation) or distantly located protein-coding genes (trans-regulation). In this realm, we looked for potential lncRNAs among the differentially expressed genes (DEGs) annotated as ‘uncharacterized proteins’ in our results and identified several differentially expressed lncRNAs (DE-lncRNAs). Furthermore, we tested the hypothesis of TAA as a novel plant growth regulator by culturing tomato cotyledons in a media containing the cytokinin 6-Benzylaminopurine (BAP) supplemented with different concentrations of TAA. This work confirms the previous effect of timentin on shoot regeneration, and is the first to report the breakdown product of ticarcillin as a novel plant growth regulator in tomato. Further studies are required to confirm similar growth regulating activity of TAA in other plant species. In addition, the identified of DEGs and DE-lncRNAs and their potential targets differentially modulated in the presence of timentin may help in elucidating the mechanisms of shoot organogenesis in tissue culture.

## Materials and methods

### Plant material and growth conditions

Seeds of tomato cultivar Roma VF were sterilized by treating with 75% ethanol for 30 seconds at room temperature and washed with sterile water for five times. The seeds were then treated with 15% sodium hypochlorite for 10 minutes, and washed five times in sterile water. The sterilized seeds were germinated on half-strength Murashige and Skoog (MS) medium *in vitro*. Cotyledons from 10-day old seedlings before first true leaves emerged were used as starting explants in all the experiments. The base and tip of the cotyledons were excised and the explants were transferred with the abaxial surface in contact with the culture medium. The basic culture medium consists of MS medium with vitamins supplemented with 30 g/L sucrose and gelled with 0.8% agar, and the pH of the medium was adjusted to 5.8 ± 0.2. Plates were maintained at a light cycle of 16/8hours day/night, temperature of 26/24°C day/night, and relative humidity of 70% and subcultured on the same media after 14 days. Observations were made on 7, 14, 21 and 28 days of growth. All experiments were conducted with at least three replicates of 10 explants each.

### Analyzing effect of timentin concentration on organogenesis

To analyze the effect of timentin concentration on root regeneration, basic culture media was supplemented with of 300 mg/L timentin. The effect of different concentrations of timentin on shoot regeneration was analyzed by supplementing the basic culture media with 1mg/L BAP and different concentrations of timentin (100, 200 and 300 mg/L).

### Plant treatments and RNA isolation

28-day old explants grown on basic culture media supplemented with 1mg/L BAP was considered control samples (CT) and those that regenerated on basic culture media supplemented with 1mg/L BAP and 300 mg/L timentin were considered as treated samples (TT). Five random explants were selected and considered as one sample. Samples from both control and treated explants were flash frozen in liquid nitrogen and total RNA was isolated using a TRIzol reagent (ThermoFisher Scientific, Cat # 15596026) based in-house protocol. RNA samples with an RNA integrity number (RIN) greater than 7 were used for library preparation. Three biological replicates each for CT and TT samples were used for RNA-seq library preparation.

### RNA-seq library preparation and data analyses

RNA-seq libraries were constructed using NEBNext Ultra RNA Library Prep Kit (Cat No. 7530) following the manufacturer’s instructions and all libraries were sequenced on Illumina NovaSeq 6000 platform (PE 150 bp chemistry). The raw fastq data generated were quality checked by FastQC (Andrews, 2010), and the adapter and low-quality regions found in the reads were trimmed using Trimmomatic v.0.39 (Bolger et al., 2014) tool (parameter: PE -threads 40 ILLUMINACLIP:adapter.txt:2:30:10 LEADING:25 TRAILING:25 SLIDINGWINDOW:4:25 MINLEN:50 TOPHRED33). Subsequently, the trimmed high-quality reads were mapped to the tomato genome (Hosmani et al., 2019) (https://phytozome-next.jgi.doe.gov/info/Slycopersicum_ITAG4_0) using HISAT2 v.2.1.0 (with parameters: -p 80 –dta –phred33 -q -S) tool (Kim et al., 2019). Moreover, alignment quality of each sample was confirmed by QualiMap v.2.2.1 (García-Alcalde et al., 2012) and aligned-read files (SAM files) were converted into sorted BAM file using SAMtools v. 1.10 (Danecek et al., 2021) (parameter: view -@ 80 -uhS and sort -@ 80). Finally, StringTie v. 2.1.3b (Pertea et al., 2015) program was used for the transcriptome assembly and read count table generation for all genes. From the read count data, gene expression based PCA and heatmap were generated using Idep v.96 (Ge et al., 2018).

### Identification of differentially expressing genes, functional annotation, and enrichment analysis

The software DESeq2 (Love et al., 2014) was used to identify DEGs. Unigenes with the expression fold change ≥1 (positive or negative) and the q-value ≤ 0.05 were considered as differentially expressed. The tomato reference genomes and related gene annotation file were downloaded from the phytozome database (https://phytozome-next.jgi.doe.gov/info/Slycopersicum_ITAG4_0) and used for gene function prediction, pfam and KEGG pathway annotation. The GO (Gene Ontology) enrichment analysis was carried out using dcGO tool (Fang and Gough, 2013) using pfam annotation. Tomato gene set based pathway enrichment analysis was performed using idep v.96 tool. The overall transcriptome analysis workflow is shown in **Figure S1**.

### Identification of differentially expressed lncRNAs and prediction of their cis/trans targets

Considering the pivotal role of lncRNA in regulation of plant growth and development, we scanned the DEGs annotated as ‘unknown or uncharacterized protein’ to mine the plausible lncRNAs. Stringent filtering criteria and multiple software tools were applied to systematically identify and classify these unannotated differentially expressed transcripts as plausible lncRNAs as described previously (Nath et al., 2021). To identify lncRNA cis-target genes, we screened for closest protein coding genes within a 10 kb window upstream and downstream of lncRNAs on same chromosome using the BEDTools v 2.25.0 program (Quinlan and Hall, 2010). To predict the trans-target gene candidates for the lncRNAs we used the RIblast algorithm to determine the mRNA- lncRNAs interaction ability (version 1.0, https://github.com/fukunagatsu/RIblast) (Fukunaga and Hamada, 2017) and those interactions showing a hybridization energy threshold of less than -30 kcals mol−1 were considered as potential lncRNA trans-targets (Jin et al., 2020).

### qRT-PCR validation of differentially expressed genes

Quantitative Real-time PCR (qRT-PCR) was used to validate the reliability of the RNA-seq results. 14 DEGs were randomly selected and verified by qRT-PCR. RNA reverse transcription was performed using QuantiTect Reverse Transcription Kit (Qiagen). Gene-specific primers were designed by Primer 3.0 software (**Table S1**). The qRT-PCR reaction mixture was prepared using PowerUp™ SYBR™ Green Master Mix (Applied Biosystems™ by Thermo Fisher Scientific, Lithuania) and qRT-PCR profiling was performed using a fluorescence quantitative instrument (StepOnePlus™ Real-Time PCR System; Applied Biosystems™). Three biological replicates and three technical replicates were used for all qRT-PCRs. Tomato actin gene (SGN-U580609) was used as internal reference. The relative gene expression level was analyzed according to the 2^−ΔΔCT^ method (Livak and Schmittgen, 2001).

### Detection of TAA

The LC-MS/MS analysis was carried out using a Shimadzu 8060 tandem mass spectrometer (Shimadzu, Japan) connected in tandem to LC-30AD (Nexera X2) ultra-high-pressure liquid chromatography (UHPLC) system, consisting of a binary pump, auto-sampler, column compartment, DAD detector and degasser. (Shimadzu, Japan). The mass spectrometer was operated in both positive and negative electrospray ionization (ESI) modes. Nitrogen was used as heating and drying gases for samples. The column used for chromatography was Zorbax Eclipse Plus C18 (Agilent Technologies, USA). The following samples were analyzed for the presence of TAA; timentin 300 mg/L solution in sterile deionized water (0 and 28 day old), tomato cotyledon grown in the presence and absence of 300 mg/L timentin in culture media (0, 14 and 28 day old).

The timentin solution in sterile deionized water was diluted further to prepare timentin 1 mg/L working solution and 10 µL was injected. The plant samples were powdered in liquid nitrogen and 0.5 g of the sample was weighed and mixed with 0.5 mL of deionized water. The sample was vortexed for 5 mins. This was followed by the addition of a solution of 5.0 mL of 1% formic acid in methanol, vortexed mixed, and centrifuged for 5 mins at 2000xg (4° C). After that, the supernatant was filtered with a disposable membrane filter of 0.45 µm pore size (Advantec, Japan). Finally, 10 µL of the filtrate was injected into the LC-MS/MS system for analysis.

### Analysis of effect of TAA and commonly used auxins on organogenesis

In order to analyze the effect of TAA on organogenesis, the basic media were supplemented with different concentrations of TAA (0.05 and 0.1 mg/L) and further compared with individual effect of widely used auxins (IAA, NAA, IBA, 2,4 D) at similar concentrations. To analyze the effects on shoot regeneration, the basic media was supplemented with 1mg/L BAP and different concentrations of TAA, IAA, NAA, IBA and 2,4 D (0.05, 0.1, 0.5, 1.0 and 2.0 mg/L) individually. Since there are no previous reports on suitable concentration ranges for TAA in tomato regeneration, concentrations at 50.0, 100.0 and 200.0 mg/L were analyzed additionally for TAA. To analyze the effect of TAA on agrobacterium growth, agrobacterium strains EHA 105 and LBA 4404 were streaked onto fresh LBA agar plates containing antibiotic rifampicin (20 mg/L) and different concentrations of TAA (100 and 300 mg/L). LBA-rifampicin plates with no additional compounds and those with 300 mg/L timentin served as a controls.

### Statistical analysis

Using R, we utilized a single factor design to conduct the statistical analysis. Explants with a visible shoot of at least 5 mm in length that continued to grow till the end of the experiment period were considered as shoot regenerating explants. We examined the variance of shoot regeneration percentage and mean number of shoots per explant with respect to the various treatments as well as the duration of the experiment using triplicate measurements. Our analysis of factorial designs employed the “aov” function, utilizing a formula that explicitly stated the relationship between the response and the factor(s) in the design. We then generated an ANOVA table by using the “anova” function. Once significant differences between at least two means were established, we performed a multiple comparison using Duncan’s Multiple Range Test (DMRT) at P = 0.05.

## Results

### Effect of timentin concentration on organogenesis

**Figure 2:**
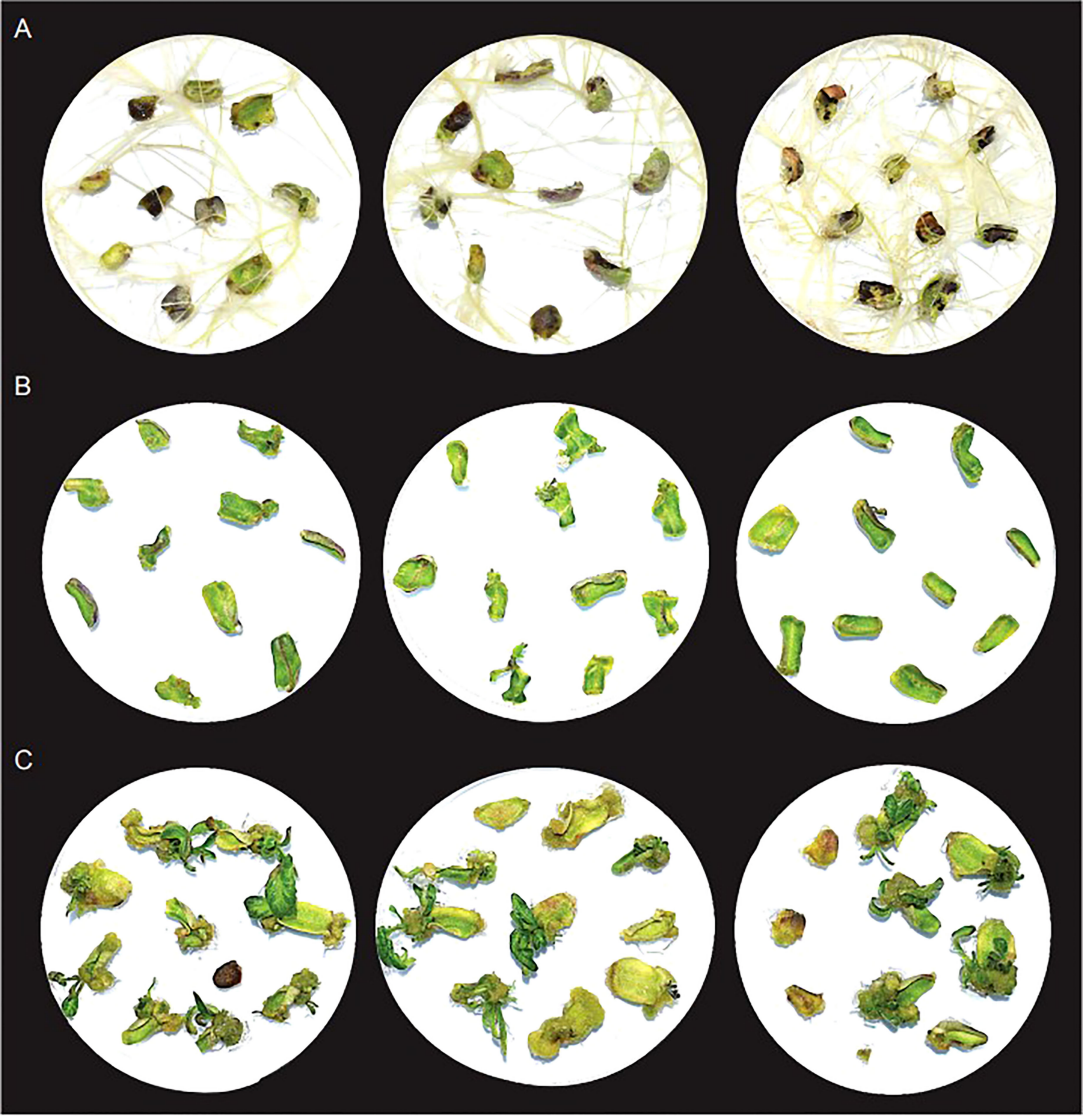
Growth of explants after 28 days in basic culture media supplemented with; **(A)** 300mg/L timentin, **(B)** 1mg/L BAP and **(C)** 1mg/L BAP and 300mg/L timentin. Three replicates for each condition is shown.

In the absence of other phytohormones, timentin aided root formation. The roots formed within 7 days of culture, and robustly grew to cover entire area of petri plates like a net by day 28 (**Figure 2**, **Table S2**). To check the effect of timentin on shoot regeneration, we used a hormone combination (basic media supplemented with 1mg/L BAP) that produced only a few tiny shoots. In this media, some of the explants showed signs of shoot regeneration after 14 days. By day 21, callus formation and tiny shoots were observed in a few of the explants. The explants increased in size only marginally by Day 28 (**Figure 2**, **Table S3**). Shoot regeneration was more pronounced when timentin was added to the medium along with BAP. Tiny needle like shoots were observable in many explants by Day 14. Shoots grew rapidly after that and at Day 28, large well-defined shoots were observed. Among the three concentrations tried, 300 mg/L timentin produced the maximum number of well-defined shoots and hence this concentration was selected for plant treatments for RNA isolation and library construction (**Figure 2**, **Table S3**). We did not analyze concentrations above 300 mg/L timentin based on our previous results from agrobacterium mediated transformations of tomato wherein using timentin at concentrations above 300 mg/L lead to formation of more callus than shoots.

### Transcriptome sequencing and assembly

To identify genes differentially expressing in the presence of timentin in tomato shoot organogenesis, RNA-seq analysis was carried out on CT (control; 3 replicates) and TT (treated; 3 replicates) samples. RNA-seq resulted in the generation of a total of 213.2 million paired-end raw reads from the 6 samples, ranging from 34.4 to 36.6 million PE reads for each sample. A total of 39.4 to 57.7 million clean reads were obtained after trimming of adapter and filtering of low-quality region. Of all the reads, 98.0 – 98.5% were aligned to the reference tomato genome (exon region alignment percentage 72.1 – 75.1). The reads with a unique alignment position on the reference sequences were much higher than those with multiple alignment positions, suggesting high-quality and accuracy, suitable for downstream gene expression and DGEs analysis (**Table S4**). A total of 1374 genes were found to be differentially expressed between CT and TT samples. Of these, 508 DEGs were upregulated and 866 DEGs were downregulated in the TT samples compared to CT samples (**Table S5**).

### GO functional annotation and KEGG pathway analysis

**Figure 3:**
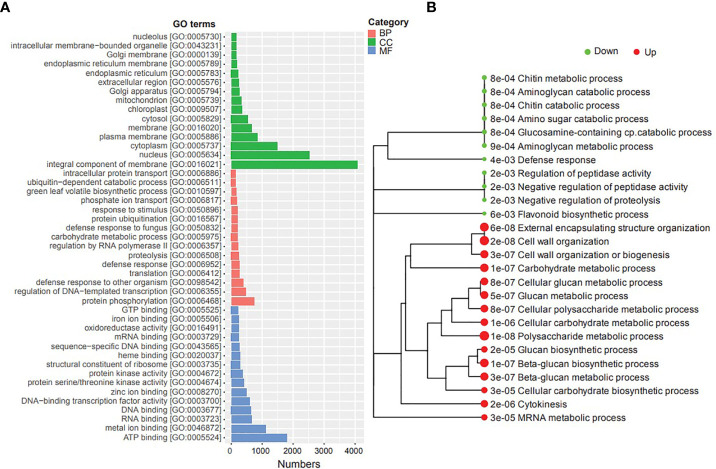
GO and KEGG pathway enrichment analyses of the DEGs identified in this study. **(A)** Top GO terms (BP, Biological Process; CC, Cellular component; MF, Molecular Function), **(B)** Metabolic pathways enriched in the transcriptome (dot size corresponds to the adjusted p-value).

We conducted Gene Ontology (GO) and Kyoto Encyclopedia of Genes and Genomes (KEGG) enrichment analyses of DEGs to gain insights into their potential functions and identify potential cellular pathways or signaling associated with timentin mediated shoot organogenesis (**Tables S6A, B**, **S7**). The GO enrichment analysis of the DEGs showed that the top GO terms associated with category ‘cellular component’ were ‘integral component of membrane’, ‘nucleus’, ‘cytoplasm’ and ‘plasma membrane’. The top GO terms associated with ‘biological processes’ were ‘protein phosphorylation’, ‘regulation of DNA templated transcription’, ‘defense response to other organism’ and ‘translation’. the top GO terms under the category ‘molecular Function’ were ‘ATP binding, RNA binding, and ‘metal ion binding’ (**Figure 3**). The KEGG analysis showed that the upregulated DEGs were significantly enriched in ‘Polysaccharide metabolic process’, ‘cell wall organization’, ‘external encapsulating structure organization’, ‘beta-glucan biosynthetic process’, ‘carbohydrate metabolic process’, ‘mRNA metabolic process’, and ‘cytokinesis’ pathways. The downregulated DEGs were significantly enriched in pathways such as ‘aminoglycan catabolic process’, ‘chitin metabolic process’, ‘negative regulation of peptidase activity’, ‘defense response’, and ‘flavonoid biosynthetic process’ (**Figure 3**).

Based on the GO terms associated with the DEGs and that of their best Arabidopsis hit, we identified DEGs associated with auxin perception, transport, signaling and metabolism, morphogenesis, and meristem formation (**Table S8**). Several key genes such as Auxin Response Factors (ARFs), GRF Interacting Factors (GIFs), Flowering Locus T (SP5G), Small auxin up-regulated RNAs etc. previously reported to be functioning in plant growth and developmental regulation were included in this.

### Identification of putative tomato lncRNAs and their targets

Initially, we annotated the DEGs based on annotation version ITAG 3.2 (https://phytozome-next.jgi.doe.gov/info/Slycopersicum_ITAG3_2), which resulted in the identification of 238 DEGs with ‘unknown/uncharacterized function’ without any assigned GO term. The stringent computational strategy led to the identification of 9 putative DE-lncRNAs with high confidence (**Table S9**). The length of DE-lncRNAs ranged from 195 bp (Solyc04g017685) to 893 bp (Solyc01g106630). Among the identified differentially modulated lncRNAs, five were upregulated and four were downregulated in shoot samples grown in the presence of timentin compared to those grown in control media. To explore the biological regulatory potential of DE-lncRNAs on the protein-coding genes, we predicted their mRNA targets based on the cis and trans-mode of interaction. We predicted a total of 27 cis-targets by screening a 10 kb window upstream and downstream of the nine DE-lncRNAs. Similarly, the RIblast program predicted 224 trans-target genes for four of the identified DE-lncRNAs (**Table S10**).

### Validation of the RNA-Seq results using qRT-PCR

To confirm the reliability of the transcriptome data, we validated the expression levels of 14 randomly selected DEGs, by qRT-PCR. The RT-qPCR results confirmed that the expression patterns of the selected genes were comparable to the results of the transcriptome analysis (**Figure S2**).

### Detection of TAA in timentin containing samples

**Figure 4:**
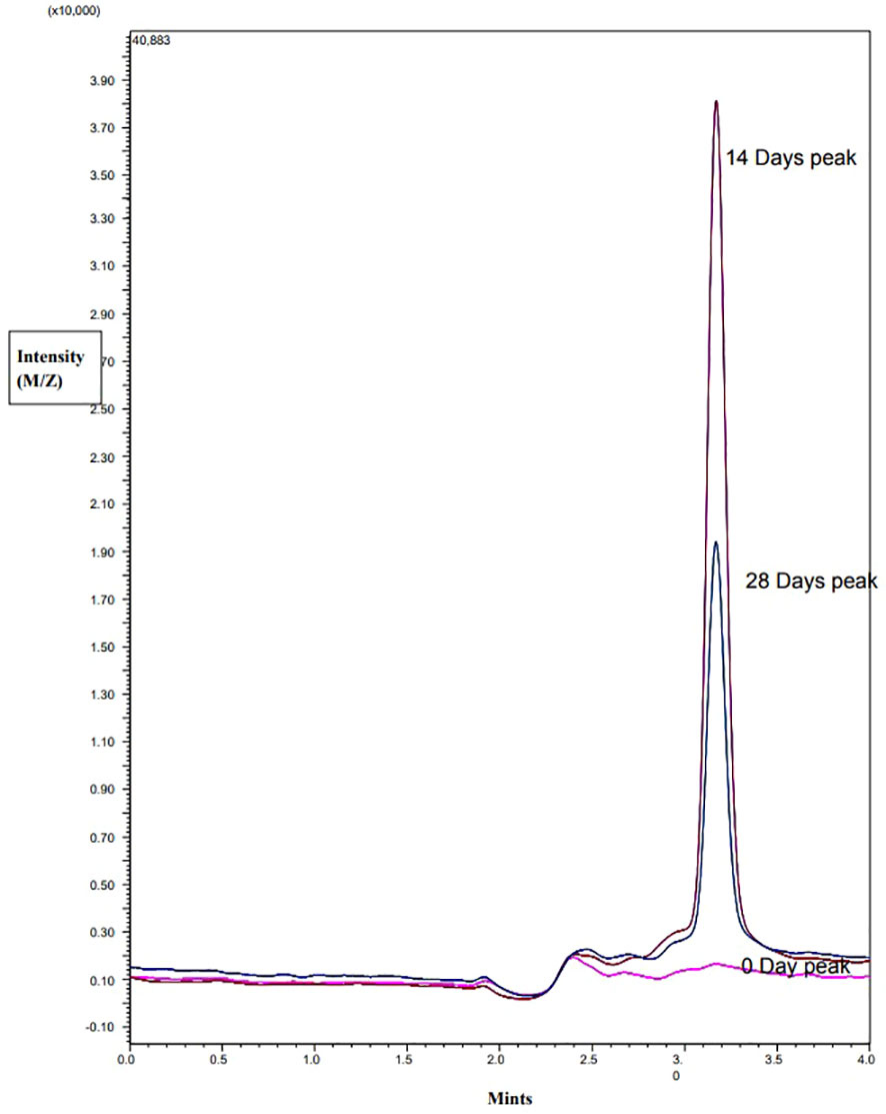
MS Chromatogram of 0, 14, or 28 days old explants grown in the presence of timentin.

The LC-MS/MS analysis revealed that TAA was not present in the freshly prepared solution of 300 µg/mL timentin in water. However, after 14 and 28 days, TAA was detected in the samples, indicating that ticarcillin had degraded into TAA during that time. In plant samples grown without timentin, no TAA was detected in samples that were 0, 14, or 28 days old. On the other hand, in samples grown with timentin, 4.6 µg/mL TAA was detected in 14-day-old samples and 2.1 µg/mL was detected in 28-day-old samples. These findings confirm that ticarcillin degrades into TAA over time, which explains its presence in the plant samples (**Figure 4**).

### Effect of TAA and commonly used auxins on organogenesis

We analyzed the individual effects of TAA and commonly used auxins, IAA, NAA, IBA and 2,4D at two different concentrations (0.05 and 0.1 mg/L) on organogenesis from tomato cotyledon explants in basic culture media in the absence of any other hormones (**Table S2**). Except 2,4 D, all the other hormones showed rapid root formation at both the concentrations within 7 days. Under both 2,4D concentrations, the explants enlarged in size and started showing signs of callus formation. Few explants showed tiny roots. By Day 14, explants on 2,4D containing media formed more callus and few of the explants showed longer roots. The explants on other compounds containing media showed rapid root growth after Day 7. The root formation pattern on TAA containing media was more similar to that on IBA containing media than others. Considering that control explants that grew on basic media without hormones also formed roots, it is possible that endogenous IAA also contributed to root formation.

**Figure 5:**
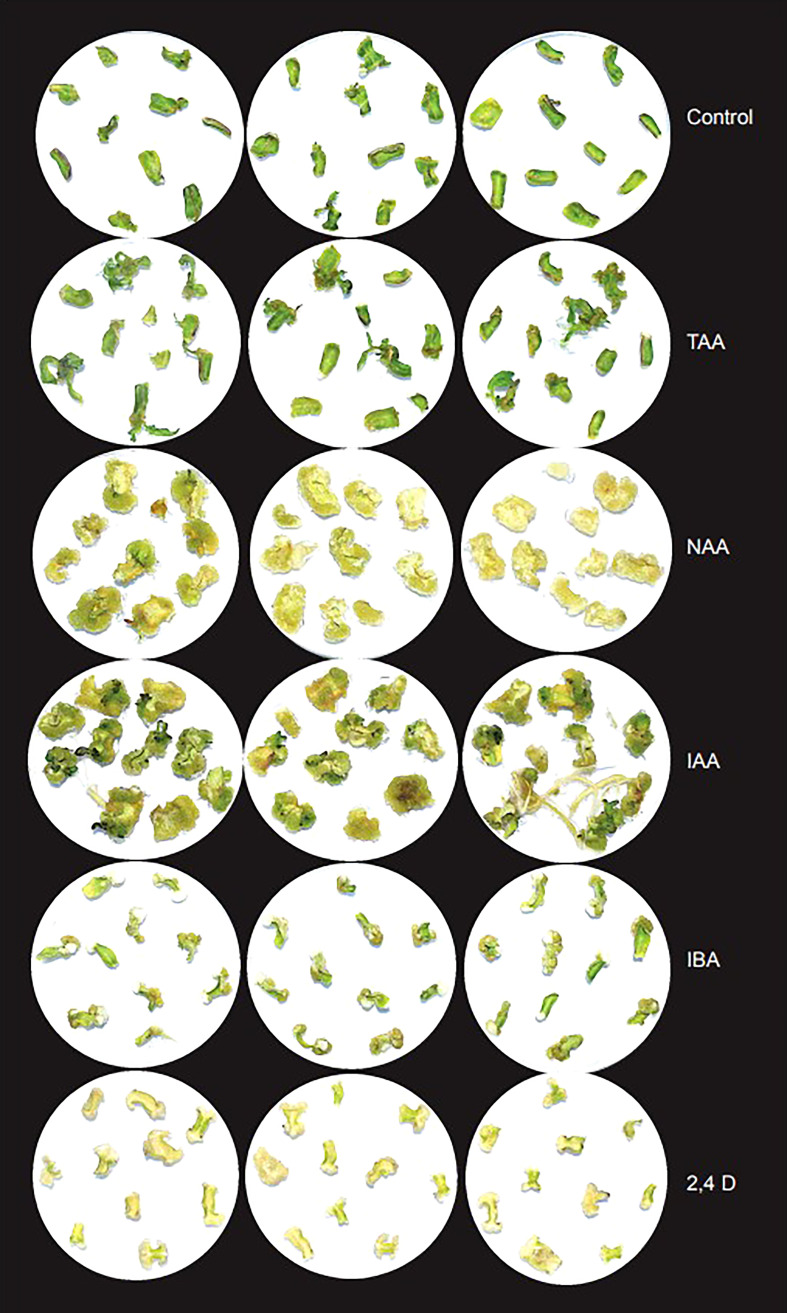
Effect of TAA on organogenesis in comparison with different auxins at 2 mg/L after 28 days in basic culture media supplemented with 1mg/L BAP.

To analyze the effect of TAA and different auxins on shoot organogenesis, we used different concentrations of TAA, IAA, NAA, IBA and 2,4D (0.05, 0.1, 0.5, 1.0 and 2.0 mg/L) along with 1mg/L BAP. In addition, we checked the effect of higher concentrations of TAA (10.0, 50.0, 100.0, 200.0 and 300.0 mg/L) on shoot organogenesis (**Figure 5**). Shoot growth was observed only at low concentration of NAA (0.05 mg/L). At this concentration, most explants produced callus, but a few produced shoots. Higher concentrations of NAA produced mostly callus by Day 28, but a few of them showed root formation. IAA showed better shoot formation than NAA at 0.05, 0.1, 0.5 and 1.0 mg/L in combination with 1mg/L BAP. The shoots at Day 28 were large and well defined. As the concentration of IAA increased, the number of explants with callus formation increased and at 2 mg/L, the plates showed mostly callus and root formation. Tiny, less defined shoots were observed too. Similar to IAA, IBA showed callus and well-defined shoot formation at lower concentrations (0.05, 0.1 and 0.5 mg/L) when used in combination with 1mg/L BAP and at higher concentrations (1.0 and 2.0 mg/L), showed mostly callus and some root formation. 2,4D did not show any shoot formation at any of the concentrations (0.05, 0.1, 0.5, 1.0 and 2.0 mg/L) when used in combination with 1mg/L BAP. The explants produced calluses at all concentrations. Lower concentrations of TAA (0.05, 0.1, 0.5, 1.0, 2.0 and 10.0 mg/L) started showing shoot formation around day 14 and many explants had well defined shoots by Day 28. TAA at 10.0 mg/L showed the maximum number of shoots. Higher concentrations of TAA (50.0, 100.0 and 200.0 mg/L) did not show any shoot or root formation, instead showed callus formation. At 300 mg/L TAA, the explants showed necrosis within 7 days and by Day 28, all explants were dead (**Table S11**; **Figure S3**). The results indicate that TAA is a weaker analog compared to the auxins tested. Analysis of effect of TAA on agrobacterium growth revealed that while timentin at 300 mg/L completely inhibited the bacterial growth, TAA did not inhibit agrobacterium growth even at high concentrations (**Figure S4**).

## Discussion

Regeneration of fertile plants from transformed somatic tissues is a major bottleneck in plant transformation experiments, especially in recalcitrant species. Tissue culture-based regeneration methods involves evaluating effectiveness of various combinations and concentrations of plant hormones (especially auxins and cytokinins) which varies depending on genotype and explants. The limited number of well-studied commonly used plant growth regulators (PGRs) is a major limitation that necessitates the identification of new PGRs, especially for those plant species and explants type, where regeneration protocols are cumbersome.

Another way to improve regeneration after transformation is to use genes that functions in reprogramming of cells and the reacquisition of an embryonic or a meristematic fate. Studies have shown that the use of DRs in plant transformation vectors increases plant regeneration after transformation. BABY BOOM (BBM), an AP2/ERF family transcription factor, can promote cell proliferation and ectopic embryo formation in cotyledons and leaves of Arabidopsis (Boutilier et al., 2002). Another DR, WUSCHEL (WUS), was found to induce embryo formation from various vegetative tissues in Arabidopsis (Zuo et al., 2002). Overexpression of Growth Regulating Factors (GRFs) from Arabidopsis (AtGRF5) positively enhanced transformation and regeneration of several monocot and dicot species, including sugar beet, canola, soybean, sunflower and maize (Kong et al., 2020). Similarly, a fusion protein combining wheat GRF4 and its cofactor GRF-INTERACTING FACTOR 1 (GIF1) significantly increased regeneration in wheat and rice (Debernardi et al., 2020). However, the use of many of these DRs is restricted by patents and thus identification of new DRs that meet the requirements of a broader range of recalcitrant species and genotypes is highly desirable. Since somatic embryogenesis depends on high concentrations of auxins, understanding the molecular mechanisms of auxin perception, transport, and signaling is essential for identifying important DRs.

We observed enhanced shoot organogenesis from tomato cotyledon explants in the presence of the antibiotic timentin. Comparative transcriptomic analysis of explants grown in the presence of timentin and those grown in media without timentin was performed to explore molecular mechanisms involved in shoot organogenesis in the presence of timentin. Particular attention was paid to genes with GO terms related to auxin perception, transport, signaling and metabolism, morphogenesis, and meristem formation. 60 DEGs were included in this category, of which 24 DEGs were upregulated and 36 were downregulated.

### DEGs involved in auxin perception, transport, signaling and metabolism

Transport of auxin across membranes is facilitated by AUX/LAX influx carriers, PIN-FORMED (PIN) efflux carriers, and ATP-binding cassette B (ABCB) transporters. In addition, the PIN-LIKES (PILS) proteins attenuate cellular auxin responses by reducing cytoplasmic IAA. Together, these proteins govern the directional cell-to-cell transport and intracellular accumulation of auxin (Larriba et al., 2021). Several auxin transporters were found to be differentially modulated in our transcriptome analysis. PILS2, an auxin efflux carrier (Solyc02g082450), is upregulated in our data, consistent with previous reports of upregulation of PILS2 in tomato hypocotyl explants during wound induced *de novo* organ formation (Larriba et al., 2021). Together with our results, this suggests a critical role of PILS2 in the distribution of auxin during organogenesis. Conversely, Solyc03g118740, encoding for SLPIN1 was downregulated in our results. Another gene involved in the maintenance of auxin flux, Solyc02g089260, encoding for ‘Auxin transport protein BIG’ was also downregulated. Solyc05g046130, coding for a ‘DUF1218 domain-containing protein’ is upregulated in our data and shows maximum similarity to Arabidopsis gene VASCULATURE COMPLEXITY AND CONNECTIVITY (VCC, AT2G32280). Expression of VCC in Arabidopsis is upregulated by auxin and the gene is reported to control the development of veins in cotyledons. VCC plays a role in the localization and stability of PIN1 (Yanagisawa et al., 2021) and is required for embryo provasculature development (Roschzttardtz et al., 2014).

One of the highly upregulated genes in our data is the tomato homolog of Arabidopsis gene At5g13750, which encodes for a ‘ZINC INDUCED FACILITATOR-LIKE’ protein (Solyc01g096720). In Arabidopsis, this gene has been reported to play major role in polar auxin transport in roots. Alternative splicing of the gene in Arabidopsis results in three transcript variants that differ in their cellular localization and function. One of the splice variants, ZIFL1.1, is involved in shootward redistribution of auxin from the apex to the elongation zone in Arabidopsis roots, whereas the other, ZIFL1.3, confers drought stress tolerance (Remy et al., 2013). In tomato, we identified two splice variants for this gene Solyc01g096720.2, and Solyc01g096720.3 which are likely to result from the selection of an alternative transcription start site downstream. Similar to Arabidopsis splice variant, the tomato splice variants might also have different functions and their role in auxin metabolism and signaling need to be elucidated further.

Another highly upregulated gene (Solyc09g091510) encodes a chalcone synthase, which is known to be involved in catalyzing the first committed step in the flavonoid biosynthetic pathway (Peer and Murphy, 2007). Flavonoids are polyphenolic compounds present in land plants and are reported to modulate auxin transport directly and indirectly by affecting the activity of auxin transporters. Silencing of CHS reduced flavonoid levels and increased auxin transport in *Medicago truncatula* roots (Wasson et al., 2006). In congruence, flavonoids have been shown as negative regulators of auxin transport *in vivo* in Arabidopsis (Brown et al., 2001). It is possible that transport of different auxins is regulated by different transport proteins and their gene expression levels reflect the presence of different auxin analogs in the tissues.

Nuclear auxin signaling pathways have three core components - the TRANSPORT INHIBITOR RESPONSE 1/AUXIN SIGNALING F‐BOX (TIR1/AFB) F‐Box proteins, the AUXIN/INDOLE‐3‐ACETIC ACID (Aux/IAA) repressor proteins, and the AUXIN RESPONSE FACTOR (ARF) transcription factors. Under low auxin conditions, the Aux/IAA repressor proteins bind to ARF transcription factors and inhibit their activity. Increasing auxin levels leads to formation of a co‐receptor complex between the Aux/IAA and the TIR1/AFB F‐box protein, resulting in ubiquitination and degradation of the Aux/IAA. After release from Aux/IAA inhibition, ARFs modulate auxin-dependent gene transcription (Powers and Strader, 2020). 22 ARFs are reported in tomato (Zouine et al., 2014). Intriguingly, we found three downregulated ARFs (Solyc12g006340, Solyc12g006350, Solyc07g043620) in our data. Solyc12g006340 (SlARF6A) was found to regulate photosynthesis and sugar accumulation, and the overexpression of the gene inhibited fruit ripening and ethylene production (Yuan et al., 2019). The roles of the three differentially expressing ARFs in our data in organogenesis is worth further investigation.

SMALL AUXIN-UP RNA (SAUR) genes encode small proteins (86–189 amino acids in Arabidopsis) and is the largest family of early auxin response genes in higher plants (Spartz et al., 2012). Tomato SAUR gene family comprises of 99 members (Wu et al., 2012). Six SAUR genes were differentially expressed in our data. Of these, Solyc03g097510 (SlSAUR38), Solyc06g072650 (SlSAUR61), Solyc01g091030 (SlSAUR1), Solyc01g096340 (SlSAUR2) and Solyc06g053260 (SlSAUR32) were upregulated whereas Solyc04g081250 (SlSAUR51) was downregulated. All six SAURs contained the auxin inducible super family domain pfam02519. Solyc06g053260, annotated as SlSAUR58 in (Wu et al., 2012), has the highest histidine content among all the SlSAUR genes and is significantly down-regulated under drought and heat stress conditions, contrary to their upregulation under salt stress. It was also up-regulated during flower and young fruit development and under IAA treatment (Wu et al., 2012). SlSAUR58 may play an important role in stamen due to the specific expression pattern in stamen and leaf (Wu et al., 2012). Our results indicate potential role for this gene in shoot organogenesis.

### DEGs involved in meristem formation and organogenesis

Solyc05g053850, coding for ‘Protein FLOWERING LOCUS T’ (SELF-PRUNING 5G, SP5G) was found to be highly upregulated in our data. SP5G mutation led to rapid flowering, suggesting its repressor role in flowering in tomatoes (Soyk et al., 2017). SELF-PRUNING (SP) gene (Solyc06g074350), which regulates growth termination was highly downregulated in our RNA-seq data. Loss-of function mutations in SP lead to accelerated termination of sympodial units, resulting in more determinate, shorter tomato plants with more synchronous flowering and fruit ripening (McGarry et al., 2020). The expression patterns of these two genes in our results indicate active vegetative growth in the samples in the timentin containing media. YABBY family (CRABS CLAW, CRC) genes have conserved roles in specifying abaxial cell fate in lateral organs such as leaves, floral organs, and ovules, and in establishing proper boundaries in meristems (Huang et al., 2013). Tomato has nine YABBY (SlYABBY) genes, two (Solyc08g079100, Solyc07g008180) of which were upregulated in our results. Floral stem cells produce a limited number of floral organs before they cease to be maintained as stem cells. AGAMOUS (AG), a MADS domain transcription factor, represses the expression of the homeodomain transcription factor WUSCHEL (WUS), which is responsible for maintaining stem cell activity in the floral meristem. The gene encoding AG (Solyc04g076680) was downregulated in our results whereas, WUS was not differentially expressed.

Differential expression of several genes involved in auxin signaling, organogenesis and meristem formation in our data indicates the effectiveness of our approach. It is worth mentioning that significant number of genes (111) were classified as uncharacterized in our results due to their missing GO terms. It is possible that several of these have important roles organogenesis. However, detailed functional analysis of these genes is necessary to elucidate their roles in timentin mediated shoot regeneration.

RNAseq analysis of plant responses to different auxins has revealed commonly as well as specifically regulated genes (Paponov et al., 2008; Fan et al., 2022). Similarly, studies have shown differential regulation of auxin transport and signaling genes to different auxins (Yamamoto and Yamamoto, 1998; Poupart and Waddell, 2000). In plants, the three central components of auxin perception and response; TIR1/AFB proteins, Aux/IAA transcriptional co-repressors, and ARF transcription factors, are encoded by multigene families (Ma et al., 2018). Different combinations of auxin receptors and transcriptional co-repressors exhibit different affinities for one another and for the kind and concentration of auxins, ultimately leading to differential gene expression in response to different auxins (Ma et al., 2018). Further studies are necessary to identify common and specific gene regulatory networks of TAA and other auxins.

### Potential lncRNAs and their targets involved in timentin mediated shoot organogenesis in tomato

We analyzed the DEGs annotated as ‘unknown/uncharacterized’ in our study to determine whether they were potential lncRNAs using several stringent criteria. lncRNAs are typically >200 nt long and have no discernible coding potential. In plants, they have been shown to function in abiotic stress responses, control of flowering time, organogenesis, photomorphogenesis, reproduction etc. (Wang and Chekanova, 2017). The stringent criteria led to the identification of nine DE-lncRNAs and their corresponding cis- and trans-target genes. Among the 27 cis targets of these lncRNAs, several were found to be involved in growth and cell wall biosynthesis, photosynthesis, gametophyte development, abiotic stress response, splicing, ribosomal biosynthesis etc. Of particular note was the prediction of mediator subunit 6 (MED 6) as a potential target of lncRNA Solyc03g121190. Mediator is a multiprotein complex that is highly conserved in eukaryotes. Mediator regulates various aspects of transcription through interactions with transcription factors, RNA polymerase II, as well as several other factors involved in transcription (Zhai and Li, 2019). The functions of MED 6 are unknown in plants and it will be interesting to unveil its function in organogenesis.

It should be noted that our studies were not tailored to identify lncRNAs and only DEGs annotated as ‘unknown/uncharacterized’ with feeble expression pattern were considered for lncRNAs prediction. In this realm, comprehensive study could shed light on the complexity of lncRNA- mediated regulation of gene expression in shoot organogenesis in the presence of timentin.

### Evaluation of plant growth regulating properties of TAA

A positive effect of β-lactam ring-containing antibiotics such as timentin, carbenicillin and penicillin G on shoot regeneration has been previously reported in species such as tomato, tobacco and sycamore (*Platanus acerifolia).* “(Nauerby et al., 1997)” investigated the effect of timentin, cefotaxime and carbenicillin on regeneration potential of tobacco leaf discs and cotyledon explants. Their results showed a positive effect of timentin on shoot regeneration from leaf discs, but carbenicillin, another β-lactam antibiotic showed no positive effect. Conversely, timentin was shown to have little effect on tobacco and Siberian elm (*Ulmus pumila* L.) shoot regeneration in another study (Cheng et al., 1998). In tomato, increased shoot regeneration from cotyledon explants was observed in the presence of 300 mg/L timentin (Costa et al., 2000). These authors suggested that auxin-like degradation products of timentin could be playing a role in this increased regeneration. (Varlamova et al., 2021) showed that timentin and Amoxicillin enhanced shoot organogenesis in tomato is concentration and genotype dependent. They suggested that the positive effects of these antibiotics on shoot organogenesis is probably due to the presence of clavulanic acid or its degradation products. These authors checked the effects of clavulanic acid on shoot regeneration using Amoxiclav®, a commercially available mix of Amoxicillin and clavulanate potassium and observed higher shoot regeneration. However, it is possible that the Amoxicillin degradation products also contributed significantly to this.

The possibility of degradation products of β-lactam antibiotics acting as auxin analogs have been examined in snapdragon (*Antirrhinum majus*) (Holford and Newbury, 1992). Their results showed that carbenicillin and penicillin G stimulate callus growth in snapdragon. HPLC, GC and GC-MS analyses showed that concentrations of these antibiotics commonly used in plant tissue culture, break down to give physiologically active levels of the auxin PAA. PAA is a naturally occurring auxin in many plant species (Korasick et al., 2013). PAA was found to induce *in vitro* shoot multiplication in *Vanilla planifolia* and chickpea (Giridhar et al., 2003; Kiran Ghanti et al., 2009).

Based on ticarcillin’s structure, we hypothesized that thiophene acetic acid might be a potential breakdown product of ticarcillin, which was further confirmed by LC-MS/MS analysis. The presence of TAA in plant samples prompted us to analyze its potential as a plant growth regulator. Our results showed enhanced shoot regeneration from tomato cotyledon explants in the presence of a range of concentrations of TAA. In the absence of other plant growth regulators, TAA influenced root formation in a manner similar to that of IBA. TAA did not show any inhibitory effects on agrobacterium growth and can be used for *in vitro* generation from transgenic explants during selection after transformation. TAA however, is unlikely to be the only degradation product of ticarcillin. Penicillin has been shown to be degraded into 6-aminopenicillanic acid (6-APA), penicillin G kalium (PGK), and PAA (Zhao et al., 2001). Further studies are necessary to evaluate the potential of other breakdown products of ticarcillin on organogenesis.

To the best of our knowledge, our results are the first of its kind to use TAA as a plant growth regulator in *in vitro* regeneration in any species. It is possible that degradation products of antibiotics such as Amoxicillin also have a potential auxin-like activity, which warrants further investigation. The discovery of new PGRs is crucial for development of efficient protocols for *in vitro* regeneration in recalcitrant species, and different hormone combinations involving TAA might prove beneficial here.

## Conclusion

Identification of novel developmental regulators and plant growth regulators is essential for developing efficient *in vitro* regeneration protocols for different plant species. To understand molecular mechanisms underlying enhanced shoot regeneration in the presence of the antibiotic timentin, we carried out a comparative transcriptome analysis of explants grown in the presence and absence of timentin. A total of 1374 genes were found to be differentially expressing in our analysis. Several key genes such as Auxin Response Factors (ARFs), GRF Interacting Factors (GIFs), Flowering Locus T (SP5G), Small auxin up-regulated RNAs etc. previously reported to be functioning in plant growth and developmental regulation were included in this. 111 genes were listed as uncharacterized in our results and did not have any GO terms associated with them. It is possible that several of these have important roles organogenesis. However, detailed functional analysis of these genes is necessary to elucidate their roles in timentin mediated shoot regeneration. We also identified 10 lncRNAs with potential roles in regulating organogenesis in tomato and identified their cis and trans targets. Additional characterization of these lncRNAs and their targets could lead to identification of novel developmental regulators. We confirmed the degradation of ticarcillin, the main component of timentin, into TAA and the uptake of TAA by tomato explants grown in the presence of timentin. We further tested the effect of TAA on organogenesis in tomato. TAA was shown to aid root regeneration from cotyledon explants and in the presence of BAP, enhanced shoot regeneration. TAA is an ideal candidate for formulating novel hormonal combinations for efficient *in vitro* regeneration of recalcitrant species. Our results are the first to analyze differential gene expression in the presence of timentin and the first to report the auxin-like effect of TAA.

## Data availability statement

The datasets presented in this study can be found in online repositories. The names of the repository/repositories and accession number(s) can be found below: NCBI SRA - PRJNA940701.

## Author contributions

Conceptualization: SG and KA. Data curation: SG, MR, MA, ME, MAN and IS. Bioinformatics analysis: NS, VSN, AKM, KMH and SG, statistical analysis: KMH and MR. Resources and supervision: KA. Writing—original draft: SG. Writing—review and editing: all authors. All authors have read and agreed to the submitted version of the manuscript. All authors contributed to the article and approved the submitted version.

## Supplementary material

Supplementary Table 5 DEGs identified in the study.

Supplementary Table 7 KEGG pathway enrichment.

Supplementary Table 8 Key differentially expressing genes.

Supplementary Table 9 List of putative tomato lncRNAs identified in this study and their cis targets.

Supplementary Table 10 List of trans targets identified for lncRNAs.
