# Supplementary material for: Ticarcillin degradation product thiophene acetic acid is a novel auxin analog that promotes organogenesis in tomato
Source: Front Plant Sci. 2023 Sep 4;14:1182074. doi: 10.3389/fpls.2023.1182074 (PMC10507259; doi:10.3389/fpls.2023.1182074)

Figure S1: Transcriptome analysis workflow

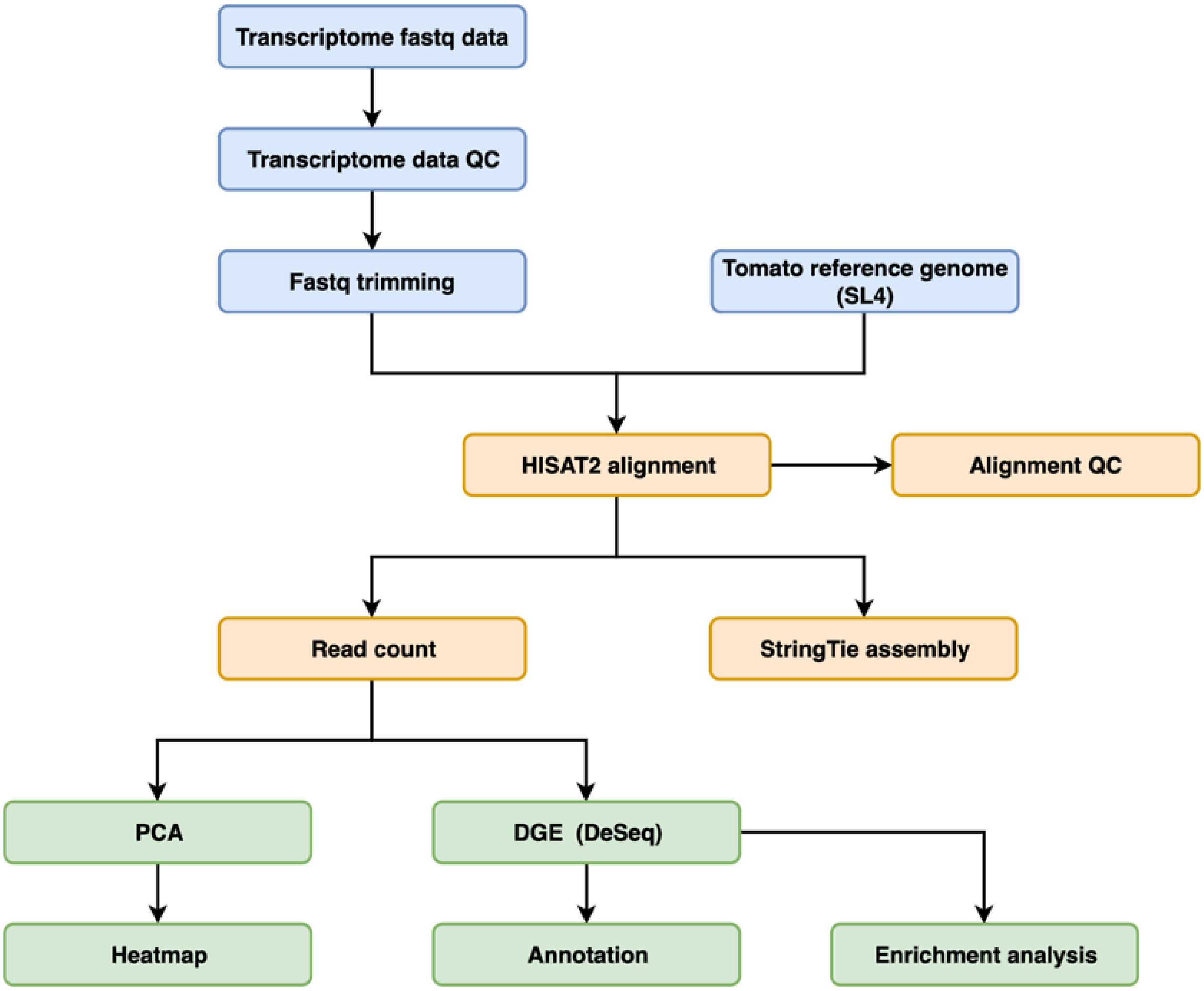

Figure S2: Validation of RNA-seq data by quantitative Real-Time PCR (qRT-PCR)

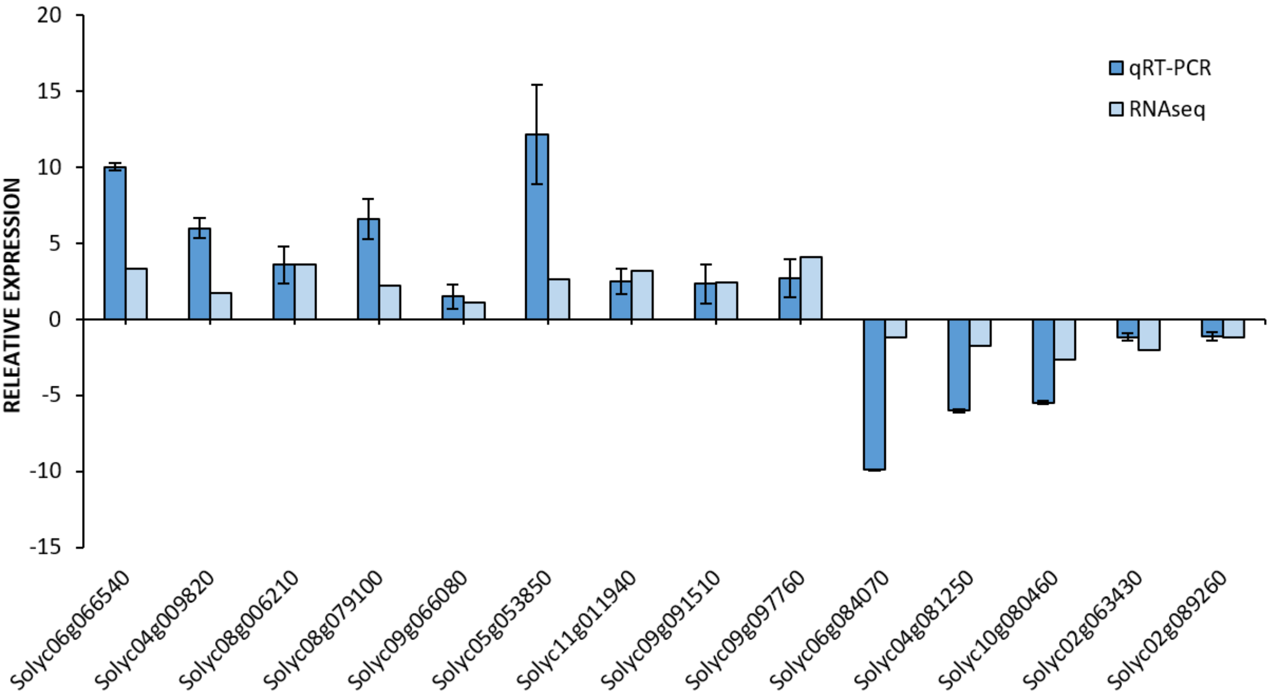

Figure S3: Effect of higher concentrations of TAA on explant growth after 28 days: the explants were grown on basic media containing 1 mg/L BAP and different concentrations of TAA. A) 10 mg/L TAA, B) 50 mg/L TAA, C) 100 mg/L TAA, D) 200 mg/L TAA and E) 300 mg/L TAA. Three replicates for each condition are shown

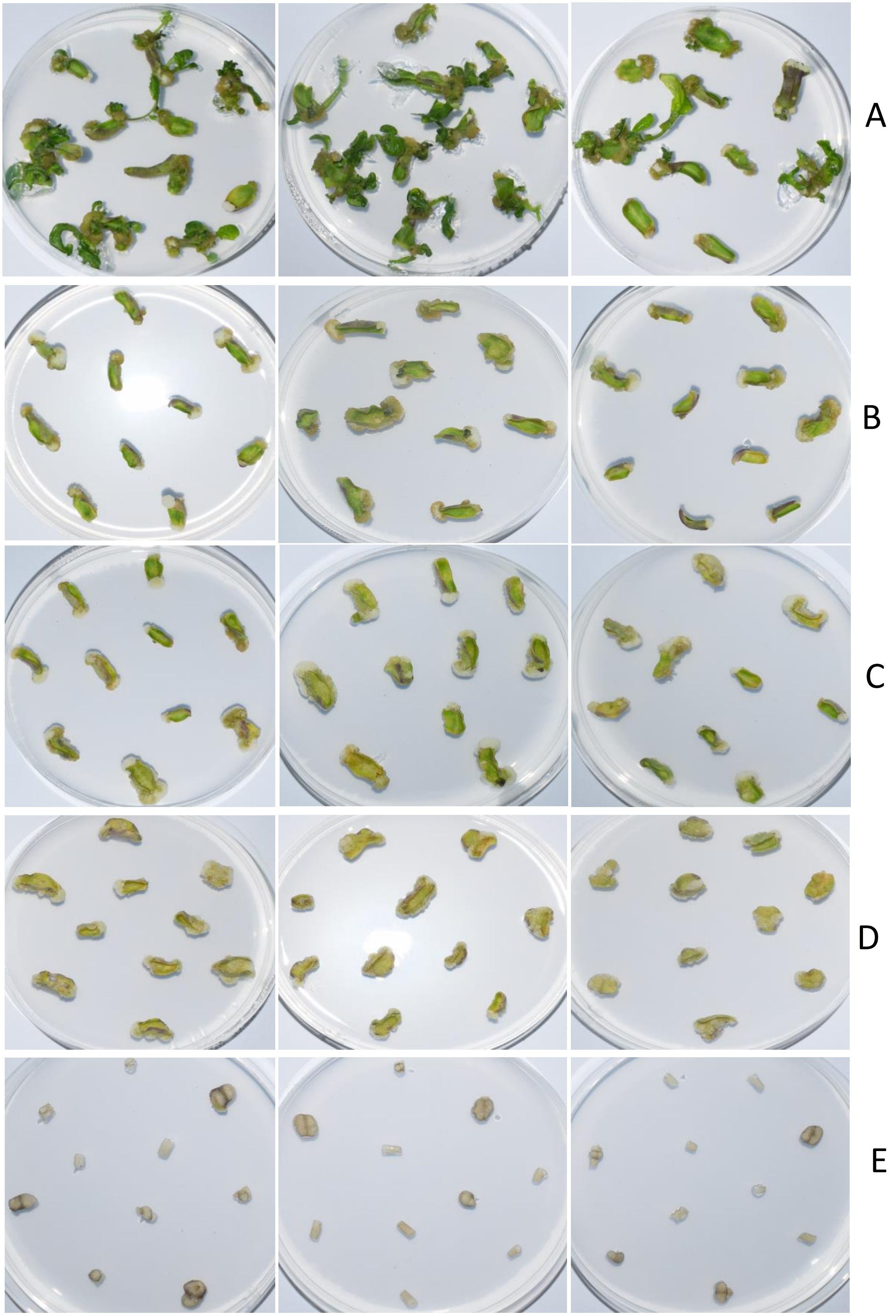

Figure S4: Effect of TAA on Agrobacterium growth. Two Agrobacterium strains EHA 105 and LBA 4404 were streaked onto LB agar media with 20 mg/L rifampicin and different concentrations of TAA and timentin. A) control plates with only rifampicin, B) 100 mg/L TAA, C) 300 mg/L TAA, D) 300 mg/L timentin. Duplicate plates are shown in each panel

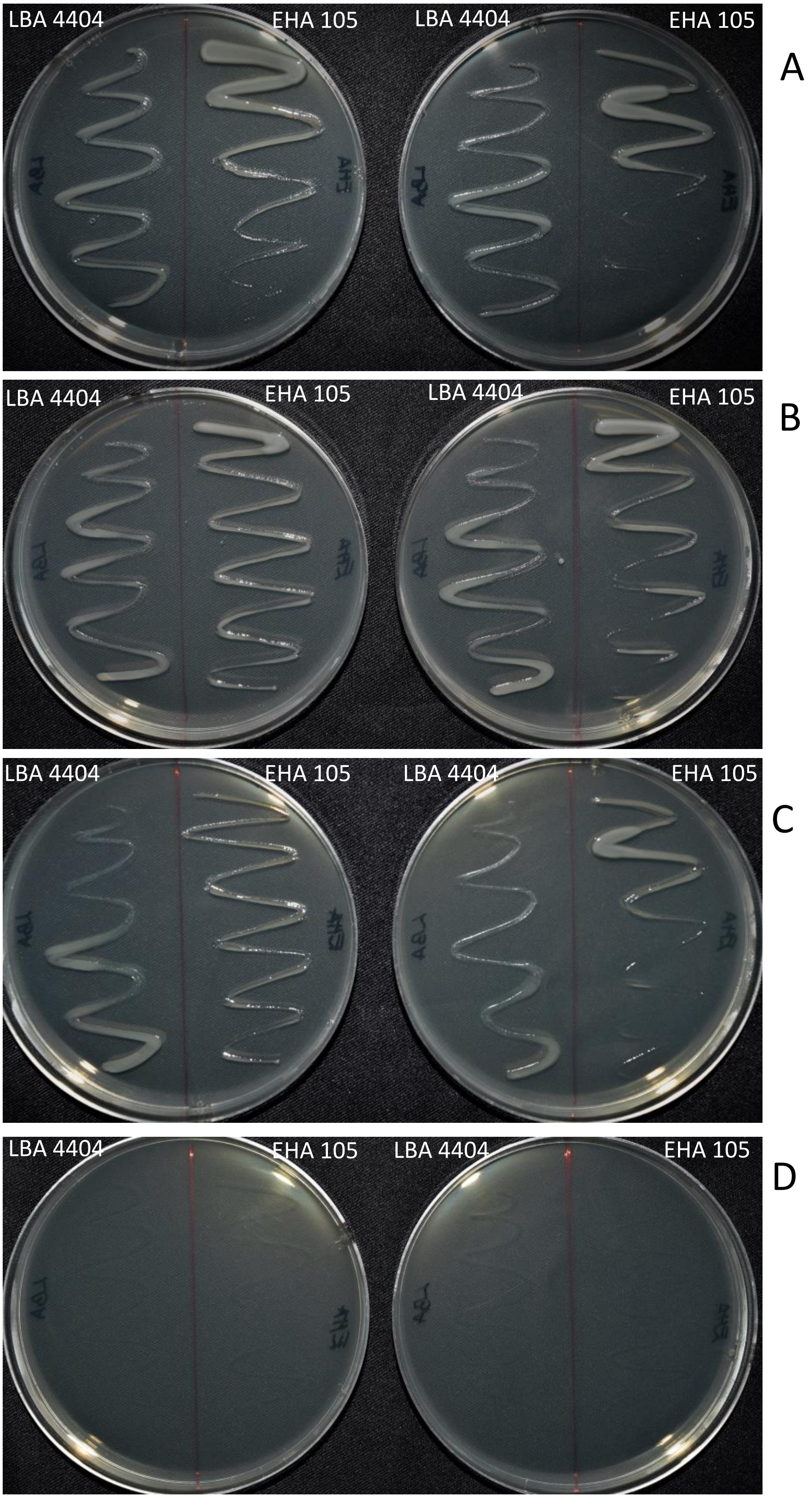

Supplement: Supplementary file 1 [file DataSheet_1.pdf]
